# An explainable covariate compartmental model for predicting the spatio-temporal patterns of dengue in Sri Lanka

**DOI:** 10.1371/journal.pcbi.1013540

**Published:** 2025-09-26

**Authors:** Yichao Liu, Peter Fransson, Julian Heidecke, Prasad Liyanage, Jonas Wallin, Joacim Rocklöv

**Affiliations:** 1 Interdisciplinary Center for Scientific Computing, Heidelberg, Germany; 2 Heidelberg Institute of Global Health, Heidelberg University, Heidelberg, Germany; 3 Department of Statistics, Lund University, Lund, Sweden; 4 Section of Sustainable Health, Department of Public Health and Clinical Medicine, Umeå University, Umeå, Sweden; Pennsylvania State University Main Campus: The Pennsylvania State University - University Park Campus, UNITED STATES OF AMERICA

## Abstract

A majority of all infectious diseases manifest some climate-sensitivity. However, many of those sensitivities are not well understood as meteorological drivers of infectious diseases co-occur with other drivers exhibiting complex non-linear influences and feedback. This makes it hard to dissect their individual contributions. Here we apply a novel deep learning Explainable AI (XAI) compartment model with covariate drivers and dynamic feedback to predict and explain the dengue incidence across Sri Lanka. We compare the compartmental Susceptible-Exposed-Infected-Recovered (SEIR) model to a deep learning model without a compartmental structure. We find that the covariate compartmental hybrid model performs better and can describe drivers of the dengue spatiotemporal incidence over time. The strongest drivers in our model in order of importance are precipitation, socio-demographics, and normalized vegetation index. The novel method demonstrated can be used to leverage known infectious disease dynamics while accounting for the influence of other drivers and different population immunity contexts. While allowing for interpretation of the covariate driver influences, the approach bridges the gap between dynamical compartmental and data driven dynamical models.

## Author summary

This research presents a novel epidemiological model that integrates climate and socioeconomic factors to better understand the spread of mosquito-borne diseases. While climate sensitivity of these diseases is well established, interactions with other drivers remain poorly understood. We integrated a compartmental model with a long short-term memory structure to disentangle contributions of precipitation, demographics, and vegetation index to dengue cases in Sri Lanka. Our model outperforms purely data-driven approaches, capturing critical non-linear dynamics like lagged climate effects and population immunity. These findings highlight the key role of precipitation and demographic factors in disease transmission and offer valuable insights for improving disease control strategies.

## Introduction

The observed and projected increase in global temperature is likely to have profound implications for climate-sensitive infectious diseases. Warmer temperatures can expand the habitats of disease-carrying vectors like mosquitoes, and increase the reproduction of insects and pathogens, such as malaria and dengue fever. Fueled by climate change, global mobility and urbanisation, dengue is one of the most rapidly spreading vector-borne diseases. Over the last 50 years, the incidence has increased 30-fold and today 3.9 billion people are at risk of infection [1,2]. Dengue is caused by any one of four closely related viral serotypes (DENV- 1, DENV-2, DENV-3, and DENV-4) of the genus Flavivirus [3]. Primary infection will lead to lifelong protective immunity to the infecting serotype and up to three months of cross-immunity against the other serotypes [1,4]. In the majority of infected cases, people do not show overt symptoms. A recent study shows that people with asymptomatic infections are approximately 80% as infectious to mosquitoes as their symptomatic counterparts and it is estimated that about 88% of infections originate from people with asymptomatic infection [5]*. Ae. albopictus* and *Ae. aegypti* are the two main vectors to transmit dengue virus. Among them, *Ae. aegypti* is the primary vector linked to the majority of dengue epidemics in the world [6], while *Ae. albopictus* is considered a secondary vector still responsible for several outbreaks around the world, it observes a greater adaptation to temperate climates [7].

The climate-sensitivity of dengue virus and vector interactions are multifaceted. Temperature is often highlighted as an important factor impacting the vector life cycle and virus transmission, affecting adult mosquito longevity, biting rate, and juvenile mosquito development and survival. Low temperatures also increase the extrinsic incubation period [3,8,9], while the relationship between temperature and mosquito life-history and transmission traits is generally observed to be unimodal, which determines temperature limits for the potential of mosquitoes to thrive and transmit diseases [10]. Mechanistic models suggest that *Ae. aegypti* transmission is optimal around 29°C and limited to the range 17–35°C [11]. In addition, various studies have shown that diurnal temperature range (DTR) also strongly affects the potential of dengue transmission [12,13]. Precipitation is another important factor in the dengue vector lifecycle, altering the availability of essential habitats for mosquito breeding. While little to moderate precipitation may increase the amount of standing water for breeding [14,15], heavy precipitation may interfere with juvenile mosquito development by washing out larvae and pupae from breeding sites [9]. There is also evidence that extreme drought can increase the risk of dengue outbreaks, by altering human water storage behavior [16]. Beyond these climatic influences, environmental factors like vegetation may also play a role, but existing studies show both positive and negative effects [17–19]. These inconsistencies in the association between climatic factors and dengue virus transmission, could be the result of confounding bias failing to adjust for other important location-specific conditions and dynamics. Association based-studies are further compounded by the frequent omission of adjustments for spatio-temporal variability in population immunity.

Socioeconomic factors are also known to exhibit strong impacts on dengue transmission. Among these, human mobility and trade may be the most important for dispersing dengue virus and vectors both at a global level and within urban communities [20,21]*.* Empirical observations have shown that mobility restrictions imposed during the COVID-19 pandemic had a substantial impact on the transmission of dengue [22,23]. The vectors as well as the virus are moved around by human mobility and trade, while otherwise the mosquitoes appear to spend the majority of their lifetime close to where they emerged, and are considered to have a limited flight range. Further on, studies show that population size and population density are positively correlated to transmission [24], while rural population, population percentage with age over 60 appear to exhibit non-monotonic relationships with transmission, potentially depending on complex and unknown factors such as age-group dependent sero-prevalence of marking variation in prior exposure to dengue virus [24,25]. Studies show that in areas with sparse populations, outbreaks tend to be of shorter duration and reach herd immunity faster compared to densely populated areas [26]. Indicators such as low education, poor housing conditions, and household income, may additionally impact transmission [24,27–29].

Integrating climatic and socioeconomic drivers into models for diseases like dengue is essential for enhancing predictions. In addition, understanding their complex influences helps guiding long-term decision making, and the development of short-term outbreak response systems. Mechanistic models excel in providing interpretability and causal insights, making them particularly valuable for scenario analyses. They are built upon our understanding of biological and ecological processes, such as vector life cycles, transmission dynamics, and host–pathogen interactions [12,30,31]. Tailoring these models to specific transmission settings typically involves calibrating parameters using observational data. To capture long-term dynamics, at least some parameters must vary over time to account for recurring patterns or shifts, such as seasonal climate variation or demographic trends. This is often achieved in a phenomenological manner, without explanatory variables, for example, by fitting sinusoids or splines [32]. With this approach, the underlying causes of the variability remain unclear and long-term predictions are unreliable. Thermal biology experiments offer an alternative by quantifying how temperature affects mosquito-pathogen traits like development rate, survival, and extrinsic incubation period. These experimentally derived relationships can be directly embedded into models, linking environmental variation to transmission potential. However, similar mechanistic understanding is harder to establish for other climatic and socioeconomic factors, which are more difficult to measure, generalize, or manipulate experimentally. Therefore, mechanistic models are often limited in capturing the full range of real-world variability and consider only a small set of explanatory drivers [33]. Moreover, even when causal relationships are established for individual covariates, their interacting or combined influences often remain poorly understood. In contrast, purely data-driven statistical models and machine learning models have been widely applied to capture complex, nonlinear relationships in high-dimensional observational data [34,35]. Statistical models aim to identify interpretable associations between variables and are often used for estimating effect sizes. Machine learning models, on the other hand, prioritize predictive performance, but often act as “black boxes” with limited interpretability and explainability. Both approaches generally lack embedded biological structure, which can increase their susceptibility to overfitting and reduce their ability to generalize across different settings.

Building hybrid models that combine mechanistic and data-driven approaches can leverage the strengths of both paradigms. Encoding domain knowledge such as serotype-specific immunity dynamics and asymptomatic transmission can not only regularize predictions but also improve interpretability and support causal reasoning. The flexibility of data-driven components enables the integration of a broader range of potential drivers and helps compensate for gaps in mechanistic understanding. To date, none of the works that have applied such hybrid models combining compartmental frameworks with deep learning methods [36–40] aimed to study the sensitivity of infectious diseases to climate variability.

**Fig 1:**
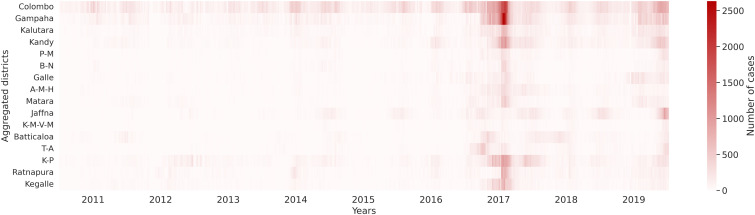
Aggregated reported cases for the 16 study districts across Sri Lanka, 2011–2019. There was a significant outbreak in 2017, with smaller outbreaks occurring in 2012, 2014, 2016 and 2019 [42]. Colombo and Gampaha generally have a higher case count than other districts partly because of the higher population number. Districts in order from top to bottom: Colombo, Gampaha, Kalutara, Kandy, Polonnaruwa-Matale (P-M), Badulla-Nuwara Eliya (B-N), Galle, Ampara-Monaragala-Hambantota (A-M-H), Matara, Jaffna, Kilinochchi-Mannar-Vavuniya-Mullaitivu (K-M-V-M), Batticaloa, Trincomalee-Anuradhapura (T-A), Kurunegala-Puttalam (K-P), Ratnapura, Kegalle. Due to the limited number of cases in a few districts, they were aggregated with neighboring districts.

In this work, we propose a new hybrid modelling method to study drivers of dengue epidemiology in Sri Lanka. While there exists a range of methods to study the spatiotemporal patterns of dengue, we wanted to study specifically here if including domain knowledge in the structure of the model by an SEIR-based approach can improve pure machine learning based forecasting of dengue. Thus, our method integrates a Long Short-Term Memory (LSTM) network, whose effectiveness in dengue prediction has been previously demonstrated [41], with a compartmental model structure. This combination enables us to incorporate immunity feedback and capture time-dependent and spatial effects in the force of infection in relation to a high-dimensional covariate space, including meteorological variability, mobility, immunity and socio-demographic conditions. We further account for the reduction in immunity with the introduction of a new strain of dengue in 2017, shifting the disease burden from DENV-1 to DENV-2 [42] due to the high population susceptibility to DENV-2. Our study is based on weekly newly reported case data from 2011 to 2019 across 24 districts, consolidated into 16 integrated districts, as illustrated in Fig 1. We compared our hybrid model with a pure LSTM machine learning model on this data and interpreted the drivers.

## Results

### SEIR-LSTM hybrid networks

**Fig 2:**
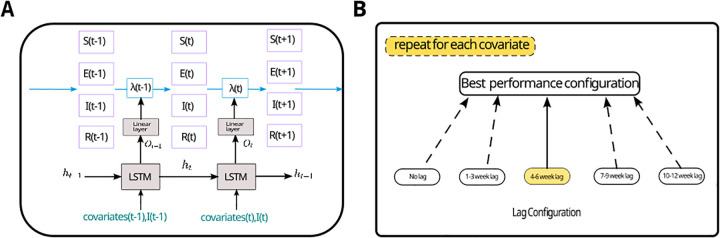
Structure of hybrid compartment model (A) and lag configuration selection (B). (A) At each time step, *t*, covariates (household income, population rate of age over 60, mean temperature, precipitation, mean NDVI) and newly infected cases, and the hidden state *h*_*t*_ from the previous time step are encoded by the LSTM model. The output, $O_{t}$, from LSTM model is then passed into a linear layer with a sigmoid activation function and amplified by a scaling coefficient to approximate the time-varying force of infection *λ*_*t*_. The compartment model with *S, E, I, R* representing susceptible, exposed, infected and recovered compartments, respectively, are driven by *λ*_*t*_. (B) We perform lag configuration selection on the validation set by shifting climate factors with different lag time steps (no lag, 1-3 week, 4-6 week, 7-9 week, 10-12 week) and get the best performance lag, worst performance lag configuration. Then we repeat the same selection for all the climate covariates. At last, we combine the best performance lag configurations of all the climate covariates to select the best lag model.

**Fig 3:**
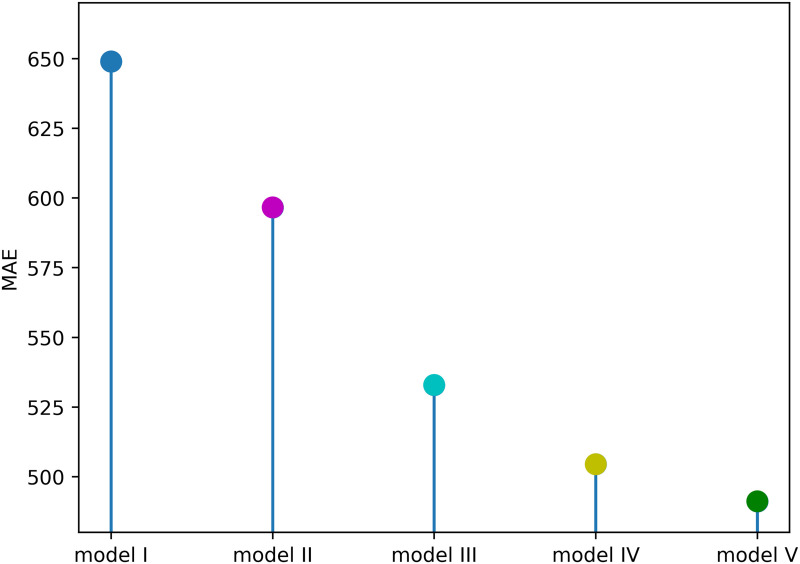
The vertical axis shows MAE values for the model configurations described in Table 2, calculated as the average result across all districts. Models I through IV are evaluated as part of the model selection process, while Models V additionally account for the strain shift and the immunity reduction observed in 2017 with the introduction of DENV-2 and are validated on 2019 prospective data.

We propose a compartmental model for mosquito-borne disease dynamics, utilizing a long short-term memory (LSTM) model to determine the force of infection, *λ*(*t*). The compartmental model employs a traditional SEIR structure for the human population. Socioeconomic and meteorology covariates, along with the current number of infected individuals, are utilized as inputs to the LSTM for estimating *λ*(*t*). The whole model structure is shown in Fig 2A and a complete list of considered covariates is presented in Table 1. With our model, we are able to track the number of newly infected individuals and changes in population immunity. We train our model on weekly dengue infection data, including both reported cases and estimated asymptomatic cases for the time period 2011–2018, covering 25 districts in Sri Lanka. The calculation details of asymptomatic cases are described in the experiment section. We validate our model using data from the year 2019 (Fig 3). In order to account for the introduction of DENV-2 in 2017, we reset the immunity level to zero at the beginning of 2017, making the whole population susceptible again. Covariate data, including socioeconomic and meteorological data, are obtained from publicly available databases and mobility data are estimated using population data and applying a radiation model [43]. Detailed information is provided in the Methods section.

**Table 1:** List of covariates.

Time-varying covariates (weekly)	Source
Mean temperature (°C)	ERA5 dataset
Precipitation (mm)	
Diurnal temperature range (DTR) (°C)	
Humidity (%)	
Max Normalized Difference Vegetation Index (NDVI)	
Mean Normalized Difference Vegetation Index (NDVI)	
Static covariates (yearly)	Calculation based on radiation model (S1 Text, Note A)
Mobility	
Population	Department of Census and Statistics website
Rural population	
Population density (per *km*^2^)	
Population proportion of age over 60	
Household income (per month)	

**Table 2:** Models evaluated during model selection and validation. Model I includes only socioeconomic covariates. Model II to V include socioeconomic factors and meteorological factors which exhibit lag effects, i.e., mean temperature, precipitation, and mean NDVI. Model V compares the full set excluding corrected covariates with introduction of the shifting strain.

Model	Description	covariates
Model I	Model with only socioeconomic covariates	Household income, population rate of age over 60
Model II	Model with the non-optimal lag configurations	Household income, population rate of age over 60, mean temperature (1–3week), precipitation (10–12week), mean NDVI (10–12week)
Model III	Model without lags	Household income, population rate of age over 60, mean temperature, precipitation, mean NDVI
Model IV	Model with optimal lag model combinations	Household income, population rate of age over 60, mean temperature (7–9week), precipitation (7–9week), mean NDVI (0 week)
Model V	Same as model IV and including the immunity by strain shift in 2017 model configuration	Household income, population rate of age over 60, mean temperature (7–9week), precipitation (7–9week), mean NDVI (0 week)

### Model selection

**Fig 4:**
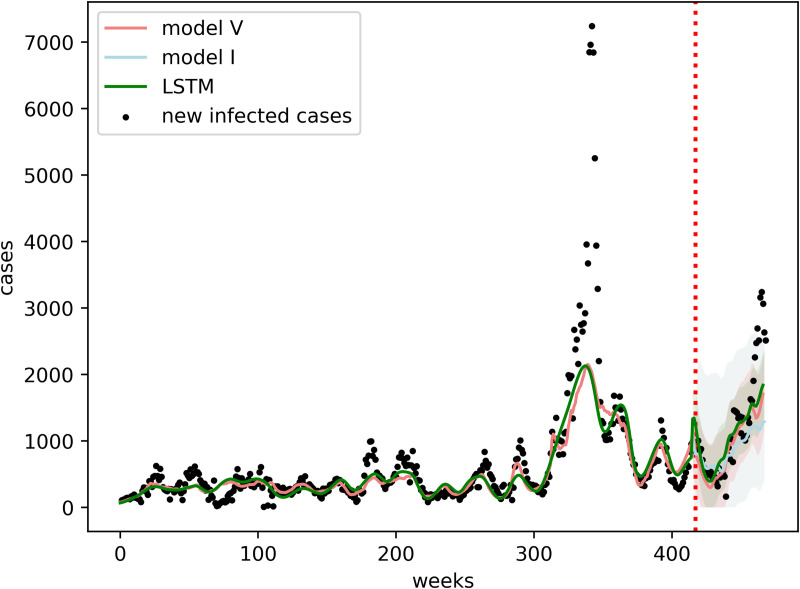
Cases fitting curves for the whole dataset over all the districts for the comparison. Y axis is the average of newly reported cases over all the districts. The Training and validation period division is indicated by the red dotted vertical line. The shaded areas represents conformal interval for model V, model I abd LSTM.

We have selected household income, population rate of age over 60, mean temperature, precipitation, and mean NDVI, as input variables for the model, with the aim of removing correlated covariates. Detailed information is provided in the Materials and Methods section, under Model Selection. We then conducted a model selection procedure to identify the best-performing configuration; the full process is also described in the same subsection. Our selection method shows that the best model includes the features precipitation, mean temperature and mean NDVI with the lag of 7–9 weeks, 7–9 weeks, 0 week, respectively (Figure B in S1 Text). Fig 3 shows the model performances of validation with different lag combinations and the advantage of accounting for changes in population immunity with the strain shift. The figure also shows that models with meteorological variables perform much better than the model without (model I) (Fig 4). Further information of the validation is shown in Figure D in S1 Text. We found that incorporating strain shift improves model performance, particularly at the beginning of the outbreak. By allowing the model to capture changes in the circulating strain and the resulting reduction in population immunity, predictive accuracy is enhanced. Detailed information of the model configurations can be found in Table 2.

### Importance analysis

**Fig 5:**
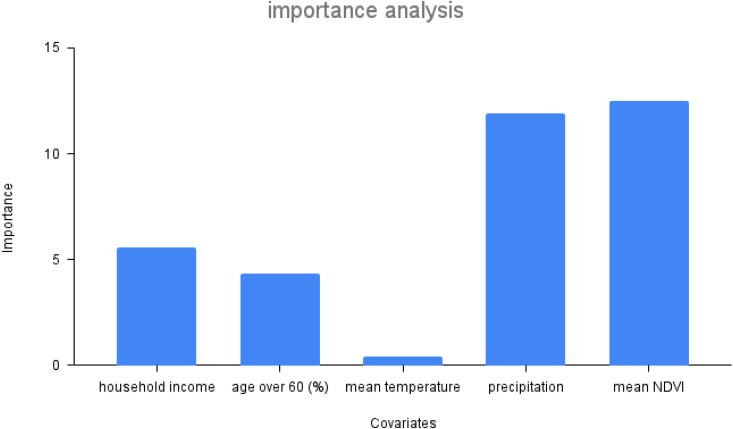
Covariate influence on the force of infection for the selected hybrid model (model V).

A deepened analysis of the optimal model - model V - is done by applying an omission method [44] for each individual covariate to understand it’s importance. In this context, importance refers to a score that indicates how useful or valuable (overall contribution) a feature was in the construction of a model. However, sensitivity is the degree to which the output of a model is affected by changes in its input variables, capturing the direction and magnitude of positive and negative effects. The details of the sensitivity analysis are described in the next subsection. The importance metric is generated by replacing one feature at each time step with the average value of the feature through the whole time from 2011 to 2018. From Fig 5, we see that precipitation, household income and NDVI appear to play the most important role in dengue transmission.

### Interpretation of covariates

**Fig 6:**
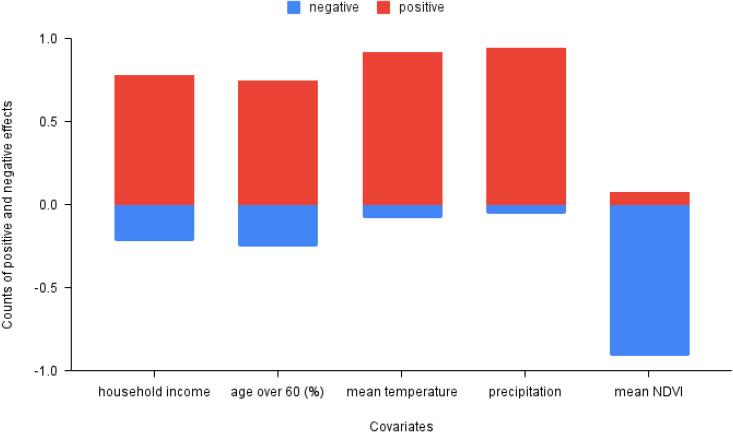
The directional impact of covariates on the cases from the selected hybrid model (V). Red color indicates a positive covariate influence and blue color indicates a negative covariate influence. The bars are the total sum of positive (red) and negative (blue) covariate influences on the outcome from a 1% change in the covariate.

In order to understand the details of how covariates affect dengue transmission positively and negatively, we use an adapted sensitivity analysis method of the covariates. Specifically, we increase each covariate 1% at each time step separately. The detail of sensitivity analysis is shown in the method section. In Fig 6, red bars show positive effects (more cases) of these covariate increases on dengue transmission, while blue bars show negative effects. From the figure, we can see that increases in mean temperature, precipitation, household income, and population percentage with age over 60 tend to have an overall positive influence. Mean NDVI, has an opposite negative influence overall, while similar to precipitation indicating a mix of negative and positive influences. More details are provided in Figure F in S1 Text Additionally, we examined the influence of mobility, as shown in Figure I in S1 Text The results indicate that mobility has an overall slight positive effect on dengue transmission but that negative effects are also observed.

### Benchmarking to an LSTM model

**Fig 7:**
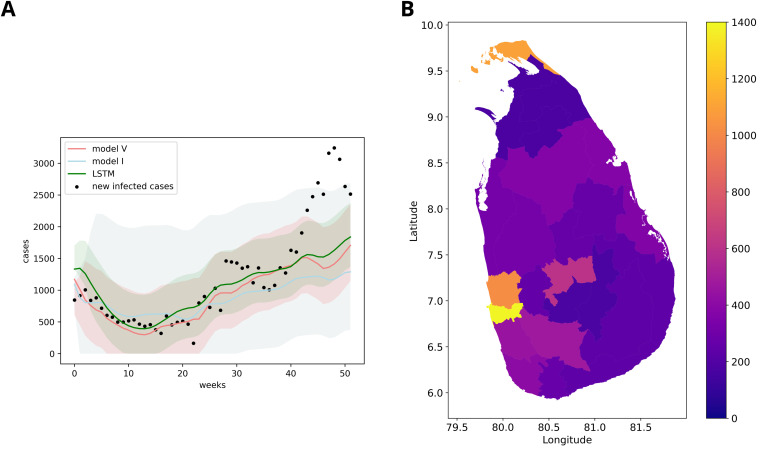
Prospective validation of the hybrid model (SEIR-LSTM) and the pure machine learning model (LSTM) on average weekly case case data for all districts in 2019 (A) and per district aggregated mean absolute error (MAE) of the SEIR-LSTM model (B). In (A) the X-axis represents the time in weeks and the Y-axis represents the newly infected cases. The blue shaded area represents the prediction interval of the LSTM model. The MAE over all time steps of the hybrid model and LSTM respectively are 491 and 527. The light red shaded area represents the confidence interval of the hybrid model (see uncertainty qualification for details). The choropleth map (B) shows the MAE of the hybrid model forecasts for each geographical district where X- and Y-axes correspond to longitude and latitude. Comparison figures as figure (A) for all the districts can be found in Fig C and Fig E in S1 Text, S3 and S5 Figs.

We compare the forecasting performance of model V with a normal non-compartmentalized LSTM model. To illustrate the predictive performance, Fig 7A displays the observed and predicted average new infected cases for all districts per week in the year 2019, which was held out from model fitting. To demonstrate the robustness of the model, Table 3 presents a comparison across additional evaluation metrics. The validation shows that the performance of the hybrid model is slightly better than the pure LSTM machine learning model in the beginning and middle of the year 2019 and that the errors increase with time in the validation period (Figure E in S1 Text). From the conformal intervals, the performance gap shows that the model is more pronounced before 30 weeks. It is shown that the LSTM model exhibits samilar uncertainty compared to the hybrid SEIR-LSTM model. The detailed information on the uncertainty quantification and the prediction interval estimation can be found in the Method section. Further information on the prospective validation is available in Fig C and Fig E in S1 Text. Overall, we observe that both models struggle to predict sudden surges, especially when moving further from model training. Fig 7B maps the forecast error geographically over the whole year of 2019. We note that the outbreak in 2019 was unusual in its temporal occurrence, mainly driven by the districts in the northern part of the country which had not been observed in prior years.

**Table 3:** Comparison on Mean Average Error (MAE), Root Mean Square Error (RMSE), and Mean Absolute Percentage Error (MAPE) of LSTM and hybrid model.

	MAE	RMSE	MAPE
LSTM	527	764	1.16
Hybrid model	491	733	0.75

## Discussion

In this study, we developed a novel hybrid model that combines LSTM algorithms and SEIR compartmental models to study drivers of dengue, including the time-varying influence of meteorological conditions. The LSTM architecture was chosen due to its effectiveness in capturing temporal dependencies, making it particularly suitable for time series data such as disease incidence [45–48]. For model training and evaluation, we used dengue case data prior to 2020 as the transmission pattern and surveillance efforts were affected by mobility restrictions associated with the COVID-19 pandemic. We select environmental drivers (including lag dependencies) and include socio-economic covariates, while accounting for serotype changes over time. The findings show that the hybrid compartmental model performs better than the pure LSTM model, while both models struggle to reliably predict the spatio-temporal occurrence of dengue cases across Sri Lanka in the validation period of 2019. One advantage of our model is that it allows us to incorporate the initial immunity and the dynamic changes in population immunity while estimating the effects of the covariate drivers. This allows us to provide insights into the complex interactions between disease dynamics, immunity, and covariates. This is also illustrated in this study by showing the better fit of a model that picks up a serotype introduction during the study period. Our findings show that beyond the importance of population immunity, household income, higher precipitation, and lower mean NDVI indicate increased dengue transmission.

In the model validation, the hybrid model shows better prediction before 40 weeks compared with the pure LSTM model (see Figure E in S1 Text). Additionally, the hybrid model exhibits slightly lower confidence in its predictions compared to LSTM, as indicated by the wider prediction intervals in Fig 7A. However, both models are struggling to make reliable predictions after 40 weeks. This may stem from the inherent difficulty of models in capturing patterns distant from the model training period, with accumulating errors over time. It may also be related to the unusual outbreak pattern observed in 2019. In fact, by 2019 the onset of the sudden surges after a 36-week period was seen only once in the training dataset. Accounting for strain shifts improves predictions before the 36-week period but these changes are relatively modest. In general, our result reveals that our hybrid model exhibits poorer predictive performance in districts with larger populations, e.g., Colombo. This trend may be attributed to the heightened variability and uncertainty inherent in datasets with higher case counts, which have the propensity to exacerbate prediction errors.

Few studies consider climate and socioeconomic factors together to dissect their respective role on the dynamics of dengue infections [24,33,49–51]. Our study shows that meteorological factors, especially precipitation, are more important than socioeconomic factors for dengue transmission in Sri Lanka. Among the socioeconomic variables considered, household income is the most important socioeconomic factor indicating that relatively high household income may lead to heightened dengue transmission (Figure F (a) in S1 Text), which seems opposite to common knowledge. This may, however, be an artifact stemming from collinearity with other factors. Specifically, higher household income areas are more often urban areas, where the population is more dense and mobility is higher. This aligns with previous studies showing that dengue risk increases in urban environments, particularly within certain population density thresholds [52]. To better understand the impact of mobility on dengue transmission, we replaced household income with mobility in the final model (Figure I in S1 Text). As is well known, major cities in Sri Lanka have higher mobility due to their larger populations. However, only a few districts in the country are densely populated, which may explain why we find both positive and negative effects of increased mobility on dengue transmission. The percentage of the population aged over 60 shows a strong impact and positive association with transmission, although its relative importance is lower than household income. This may reflect the general impact of age-related vulnerability at the population level, with recent years seeing increases of infection in older age groups.

Among the environmental factors, we find that precipitation and NDVI are the most important factors for dengue transmission. Precipitation exhibits mainly a positive effect. This is likely because it provides critical water for mosquito breeding. Interestingly, we do not find that heavy rainfall has much of a negative effect on transmission (Figure F (d) in S1 Text). Previous studies have documented that heavy rain create more breeding sites and deeper water, which will benefit mosquito breeding [53]. Based on the correlation matrix (Figure A in S1 Text), humidity is another important factor but it was not included here since it’s highly correlated to precipitation. However, high humidity together with massive precipitation can lead to increase in mosquitoes longevity, feeding activity, and in breeding conditions [54,55]. Previous studies rarely suggested NDVI as an important driver of dengue transmission [6,33,56]. However, wee here we assume NDVI to serve as a proxy for land use and human settlement density, opposite to its normal indication. We use it in this way because *Ae. aegypti* is a highly anthropophilic species that prefers human blood and mainly breeds in close vicinity to human settlements in artificial breeding sites such as discarded tires and covered containers [57]. Although *Ae. albopictus* has a wider ecological plasticity with respect to breeding sites and blood feeding behavior [58], but it is equally capable of breeding in man-made habitats. Our results show that mean NDVI is the most important covariate among the environmental factors considered (Fig 5), showing a negative impact on dengue transmission (Fig 6). In addition, population and topography specifications in different districts magnify the discrepancy of NDVI effects geographically. Urban areas like Colombo and Gampaha are less vegetated and densely populated, which likely contribute to more contact among humans and dengue vectors. The other districts are densely vegetated rural areas, and are more conducive to *Ae. albopictus* for dengue virus transmission [59,60]. Finally, mean temperature showed a positive contribution to transmission, although less impactful than rainfall.

Previous studies have taken alternative routes and documented forecast skill in other settings using LSTM, Bayesian spatio-temporal approximation method (INLA), decision tree models, multi-layer perceptron, support vector machine and Bayesian networks [34,35]. In addition, several works share conceptual similarities with ours. Arik *et al.* [36] have developed a hybrid model that encodes covariates into a compartmental model for COVID-19. Delli *et al.* [37] use a feed-forward neural network to predict the residuals of the SEIR model. Rahmadani *et al.* [39] use a deep neural network to minimize the gap between real COVID-19 data and the estimation of their meta-population model. To the best of our knowledge, most hybrid models have been designed specifically for COVID-19 and lack the integration of covariates and interpretability. In contrast, our model is uniquely tailored for vector-borne diseases, incorporating considerations for changes in vector-virus dynamics and population immunity. We found that perturbation-based interpretation methods are effective for complex black-box models, as they do not require any specialized modifications and preserve the integrity of the model structure. Moving forward, we believe it is essential to incorporate mosquito information into the hybrid model as prior knowledge, potentially by extending the compartmental structure by introducing a refined model for mosquito population dynamics or encoding mosquito life cycle indices. Future research should investigate the added value of the approach taken here more exhaustively by comparing it with a wider range of alternative modelling approaches.

Our method can readily be extended to other vector-borne and environmentally driven diseases, and also to a broader range of infectious diseases. While some mechanistic understanding of transmission exists for diseases like Zika, chikungunya, or cholera, this knowledge is often incomplete, particularly regarding spillover and environment driven dynamics. In such cases, machine learning and neural network models can play a critical role by capturing complex, nonlinear patterns in the data that are difficult to encode explicitly in compartmental models.

In the interpretation aspects, our model could quantitatively provide an interpretation of covariates, including conditions of meteorological, socioeconomic conditions and NDVI. The estimated lag time also provides information of relevance for early warning of dengue transmission, but the models need further development and validation for serving as an early warning tool. Nonetheless, the adapted explainable method provides a step towards data-driven and covariate dependent interpretable SEIR dengue models.

A limitation of our study is that all dengue and socioeconomic data was aggregated at district level, which prevents us from elucidating important drivers at smaller spatial scales. A further limitation was the approximation and interpolation of the population immunity from non-complete serological measurements, and the inclusion of modeled mobility data rather than observed. Due to its high correlation with other covariates, particularly household income, mobility was not retained in the final model. However, its effect is captured through these correlated variables. Although this simplification improves model interpretability, we acknowledge that it may come at the cost of reduced predictive performance. Moreover, the scarcity of data, along with the fact that immunity effects only become apparent over extended periods, constrains the accuracy of long-term predictions.

## Materials and methods

For each district we impose a compartmental model, where the force of infection is driven by a long short term memory model. In this section we describe in detail the model building. We first introduce a compartmental model of mosquito-borne disease, then, we describe the encoding of covariates into the compartmental model. Finally, we explain an end-to-end training of the model and how the developed model is compared with a pure machine learning method.

### Compartmental model

Compartmental models are traditional mathematical methods to describe the dynamics of infectious diseases. It separates a population into several compartments, e.g., S (susceptible), E (exposed), I (infectious), R (recovered).

For the human population, we use a SEIR-model, see Equation (1). In Equation (1), *α* is the average daily vector biting rate, *b*_*h*_ is the probability of vector to human transmission per bite, *A*_*I*_ is the infectious adult female mosquito population, ***S*** denotes the susceptible population, ***E*** denotes the exposed population, ***I*** denotes the infectious population, ***R*** denotes recovered population, *N* is the total human population, which we assume to be constant, *γ* is the recovery rate, *ω* is the progression rate from the exposed to the infectious stage [12,30,61].


$$\frac{dS}{dt}=-\lambda S$$

$$\frac{dE}{dt}=\lambda S-\omega E$$

$$\frac{dI}{dt}=\omega E-\gamma I$$

$$\frac{dR}{dt}=\gamma I$$

$$\lambda =\frac{\alpha b_{h}A_{I}}{N}$$

N=S+E+I+R (1)

Since mosquito data is not available for each district in Sri Lanka, the hybrid model was developed to help us drive the dynamics by only using the information of climatic and socio-economic covariates and new infected human cases. The vectors are thus not directly modeled. Instead changes in the number of infectious adult mosquitoes are indirectly captured through our covariate-driven LSTM model of the force of infection.

In order to model the imported strain in 2017 [62,63], we reset the compartment model immunity when the new dengue strain is introduced. We assume that both strains have the same transmission rate, but the human population has zero immunity due to the shifting strain at the beginning of 2017. To simplify the problem, we ignore the potential effects of antibody-dependent enhancement.

### Hybrid model

We approximate the force of infection, $\lambda (t)=ab_{h}\frac{A_{I}}{N}\epsilon R^{n\times \text{1}}$with an encoder using the size of infectious population ***I*** $\epsilon R^{n\times \text{1}\times T}$and covariates, ***Z*** $\epsilon R^{n\times d\times T}$**,** as input, in Equation (2), where *n* is the number of aggregated districts, *d* is the number of covariates, and *T* is the whole time window size. Specifically, we use a long short-term memory (LSTM) network [64] as an encoder to estimate the force of infection at each time step. A detailed description of LSTM is provided in Note A in S1 Text. We assume the estimated new infected cases are close to the real newly infected cases and normalize them by min and max normalization. Here, *σ* is the standard sigmoid function, $\lambda_{Max}$ and $\lambda_{Min}$ are lower and upper bounds of the force of infection, respectively (For all simulations $\lambda_{Max}$ = 0 and $\lambda_{Max}$=0.01 for simplicity). $W\epsilon R^{\text{3}\times \text{1}}$ is the weight to encode the output feature from LSTM to dimension 1. This interval was selected based on manual trial and error simulations.


$$\lambda (t):=ab_{h}\frac{A_{I}}{N}\approx encoder(Z(t),I(t))=\left[\lambda_{Min}+(\lambda_{Max}-\lambda_{Min})\cdot \sigma (W^{T}LSTM(Z(t),I(t)))\right]$$ (2)

Finally, we discretize our compartmental model by applying Euler’s method with a time step length of one week according to the weekly dengue case data, see Note B in S1 Text. For a complete description including seroprevalence estimates for the initial conditions, see Figure H in S1 Text. The initial values are described in the experiments section.

### End-to-end training

We utilize Symmetric Mean Absolute Percentage Error (SMAPE) as the loss function (Equation 3) for the hybrid model to supervise the training.


$$\begin{array}{c} {Loss}_{lstm}=\frac{\text{1}}{T}\sum {\mathrm{\backslash nolimits}}_{t=\text{0}}^{T}\frac{\text{1}}{N}\sum {\mathrm{\backslash nolimits}}_{j=\text{0}}^{N}\frac{\text{2}\left|I_{j}^{lstm}(t)-I_{j}^{label}(t)\right|}{\left|I_{j}^{lstm}(t)\right|+\left|I_{j}^{label}(t)\right|} \end{array}$$ (3)

where *t* denotes the week number, *j* denotes the district number. $I_{j}^{lstm}$ denotes the estimated newly infected cases, i.e., $I^{lstm}=\omega E$. $I_{j}^{label}$ are the adjusted new infected cases (reported and unreported new infected cases). In the experiments, we set the recovery rate *γ* as 1 week^-1^ and the progression rate *ω* equal to 7/10 week^-1^, according to WHO dengue guidance [1]. The loss function for the overall network is:


$$Loss={Loss}_{lstm}+\eta L_{\text{2}},$$ (4)

where *L*_2_ is the L2 norm for LSTM parameters and *η* is a regularization parameter and set to 10e-6 based on trial and error, balancing the loss term and avoiding model overfitting.

### Model selection

A preliminary Spearman correlation analysis of covariates (climate factors without lags) indicated that several variables exhibit strong correlation, the results are compiled in Figure A in S1 Text. To avoid multicollinearity, we have selected the covariates, household income, population rate of age over 60, mean temperature, precipitation, and mean NDVI, as inputs for the model, ensuring that the correlation between covariates did not exceed the preset limit of 0.6.

The model selection was performed in four steps. As the first step, we have defined a base-level model, model I, by only including socio-economic factors. Then, we define the lags of different meteorological factors as affecting model performance substantially for up to 12 weeks. To account for this, we divide the 12 weeks into 4 average lag strata groups by 3 week intervals (1–3, 4–6, 7–9, 10–12). Once we have found the best lag for one covariate we use that configuration for the next covariate, i.e., we increase the model step by step. Thus, we end up with a final model with the best lag configuration. We repeat this for each meteorological covariate as shown in flowchart Fig 2B based on the lowest MAE and highest MAE setting from last selection. We then trained a final selected lag model with the lowest MAE lag configuration (model IV). Similarly, we selected the highest MAE lag configuration to obtain model II and trained a model without lag configuration (model III). Then by comparing these combined models, we select the best-performing model among them as the third step. In the fourth step, we validate our selected model accounting for the 2017 DENV-2 strain shift (model V) by comparing it with the best-performing model among those that do not account for the strain shift (model IV).

### Machine learning for comparison

We compare our hybrid model to a pure LSTM model. Instead of using LSTM as an encoder to encode covariates to the force of infection as in the hybrid model, we utilized normalized lagged covariates as model V and estimated normalized new infected cases as input, and normalized new infected cases as target for the output, respectively. Our LSTM model consisted of four LSTM cell layers and one fully connected layer, with the same structure as the LSTM in the hybrid model.

### Uncertainty quantification

We have adopted conformal inference for the uncertainty measurement as it is widely used in time-series prediction [65,66]. The core concept involves training a regression model on the training data and then using the residuals (difference between predictions and target values) from a held-out validation set to estimate the prediction intervals in future predictions for a given confidence level. Here, we use a significance level *α* = 0.05 for a 95% prediction interval in the forecast area and a sliding window of most recent *W* residuals equal to a year to estimate the quantile boundaries.

### Data

We utilized Sri Lanka dengue data for our training, which spanned 8 years (2011–2018) and consisted of weekly data from 25 districts. The reported new infected cases served as the ground truth, which was obtained from the epidemiology unit website of Sri Lanka [67]. Based on the asymptomatic rate statistics [68], we estimate that the real infected cases are 11 times higher than the newly reported cases. This expansion factor is used to adjust the ground truth. The inputs comprised time-varying covariates like mean temperature, and static covariates like household income, which remained constant throughout the year. The static data is from the Department of Census and Statistics website [69], while time-varying data is aggregated weekly from ERA5 dataset [70]. Table 1 contains a list of these covariates, and an example plot of the time-varying covariates for the Colombo district can be found in Fig G and Table A in S1 Text. We have calculated the correlation between the covariates and removed highly correlated covariates. The detailed information of correlation of the covariates can be found in Figure A in S1 Text. We used the district populations as initial susceptible individuals (subtracting the infected cases and immune population as detailed below). The immune population was set as the initial recovered population, while the exposed population was initialized to zero. For the initial infected population, we used new infected cases calculated based on the reported cases in the first week and listed them in Table B in S1 Text. The statistics of seroprevalence used to calculate the immune population of each district are applied in the model and shown in Fig H and Table C in S1 Text, according to the Department of Census and Statistics website [69]. In 2017, one model included a reset of the recovered and immune population with the introduction of a new strain. We selected 416 weeks of data from 2011 to 2018 as the training data, and another 52 weeks from 2019 as validation data. Since some districts have very few cases and populations below 500,000, we decided to aggregate some of the districts based on their geographic location, population and reported case number. Districts that were neighbors, had populations lower than 500,000, or had reported case numbers lower than 20 per district were aggregated. The 25 districts were aggregated into 16, as illustrated in Fig 1.

### Importance Analysis (IA) for all the covariates

In order to evaluate the importance of each covariate, an importance analysis was conducted over all districts (*d*) and time-steps (*t*). The adapted omission method [44] was employed, which defines the importance of a features $x_{i}\;(IA(X_{i}))$, for a model Y=M(X), with input vector *X* as:


$$\begin{array}{c} IA(X_{i})=\frac{\text{1}}{T}\sum {\mathrm{\backslash nolimits}}_{t=\text{0}}^{T}\left|M(X_{t})-M(X_{\underset{―}{i},t})\right|. \end{array}$$ (5)

Here, *X* consists of *k* features and $X_{\underset{―}{i},t}$ means the replacement of the *i*^*th*^ input feature at time *t* by the average of the *i*^*th*^ input feature *X*_*t*_. $IA(X_{i})$ stands for the importance of covariate *X*_*i*_. To calculate the importance of each covariate more accurately, we scale the importance by multiplying $IA(X_{i})$ by the standard deviation of the corresponding feature *σ*_*i*_, i.e., $IA(X_{i})\sigma_{i}$ [71]. The covariates for training are used for importance analysis. Thus, we are able to compare both time-varying and static covariates to determine their global importance.

### Directional effects of covariates

To give a more indepth interpretation of each covariates, we adapted the omission method to calculate positive and negative effects of covariates on the incidence number. Specifically, we define a sensitivity analysis as below:


$$\begin{array}{c} SA(X_{i,t})=\frac{\text{1}}{n}\sum {\mathrm{\backslash nolimits}}_{j=t+\text{1}}^{t+n}(M(\Delta X_{i,j})-M(X_{j})) \end{array}$$ (6)

where, $\Delta X_{i,j}$ denotes the input vector at time *j*, where the *i*^*th*^ input feature is increased, i.e., $\Delta X_{i,j}$ is same as *X*_*j*_ but where the *i*^*th*^ input feature has been increased. We increase the *i*^*th*^ input feature by 1%, compared to the corresponding value in *X*_*j*_. In our simulations we set *n* = 4. For each covariate *i*, we calculate $SA(X_{i,t})$ at each timestep *t* and count the number of positive and negative effects. A positive effect means that an increase in $X_{i,t}$ leads to an increase in the incidence number ($SA\left(X_{i,t}\right)>\text{0}$), while a negative effect means the opposite. The presence of both positive and negative effects over time for a single covariate suggests a non-monotonic relationship between the covariate and transmission.

### Model comparison implementation details

In order to compare the forecasting performance at different time steps, the trained hybrid model and LSTM model are compared on the validation dataset. Specifically, the hybrid model was trained on 8 years of data and predicted on 1 year. The pure machine learning LSTM model adopts the same strategy for prediction.

We implemented the method using PyTorch [72]. For training, we utilized the Adam optimizer [73] with a learning rate of 0.01 using a cosine annealing schedule and trained for 4500 epochs. During validation, as there were no reported cases available for the model, we used the predicted newly infected cases as the input for the next time-step. The compared LSTM model utilized the same network structure to ensure fairness in the comparison. After tuning hyper-parameters, we choose weight decay $\eta =\text{5}\times {\text{1}\text{0}}^{-\text{4}}$ for both models. We trained the model on a GeForce RTX 2080 Ti GPU with 11GB of memory.

## Supporting information

S1 Text **Supplementary Note A in S1 Text.** The LSTM model. Provides detailed description of LSTM, with Equations S1–S6. **Supplementary Note B in S1 Text.** Discretized compartmental model by Euler method. Provides detailed description of compartmental model, with Equations S7. **Supplementary Note C in S1 Text.** Encoding mobility. Provides detailed description of mobility, with Equations S8, S9. **Fig A in S1 Text.** (a) Spearman correlation coefficients of covariates. (b) p value of correlation coefficients of all the covariates. **Fig B in S1 Text. Model selection of climate covariates for different lag groups.** The vertical axis shows MAE values for different lag models described in the model selection section, calculated as the average result across all districts. **Fig C in S1 Text. Cases prediction for each integrated districts.** Y axis is the cases and X axis is time in weeks. Orange line represents LSTM, pink line represents hybrid model and dark points (label) represents newly reported cases data. **Fig D in S1 Text. Mean Absolute Error (MAE) calculated on validate data over all the districts for the comparison of with and without strain shift.** Y axis is the average MAE of newly reported cases over all the districts. MAE over all time steps of the model introducing the shifting strain(model V) and the model without introducing shifting strain(model IV) are **491** and **519**. **Fig E in S1 Text. Mean Absolute Error (MAE) calculated on validate data over all the districts.** Y axis is average MAE of newly reported cases over all the districts. MAE over all time steps of the model introducing the shifting strain and the model without introducing shifting strain are **491** and **538**. **Fig F in S1 Text. Interpretation of important covariates.** Each point in the figure represents the change of output of the model at different times and different districts, when the input was increased by 1%. The details of our adapted sensitivity analysis can be found in the method section. The X-axis represents value of covariates. Y-axis represents the increment by our sensitivity analysis. **Fig G in S1 Text.** Climate covariates of years from 2011 to 2019 for Colombo**Fig H in S1 Text.** Map of assumed seroprevalence of Sri Lanka at the start of year 2011. **Fig I in S1 Text.** The directional impact of covariates on the cases for population percentage of age over 60, mobility, mean temperature, precipitation and mean NDVI. **Table A in S1 Text.** Example of socioeconomic data for Colombo district **Table B in S1 Text.** Population of different integrated districts in 2011 as initial value of S and newly reported cases in the first week of 2011 as initial value of I. **Table C in S1 Text.** Seroprevalence estimates of Siri Lanka based on sero-prevalence surveys and model interpolation.
(DOCX)
