# Supplementary material for: An explainable covariate compartmental model for predicting the spatio-temporal patterns of dengue in Sri Lanka
Source: PLoS Comput Biol. 2025 Sep 26;21(9):e1013540. doi: 10.1371/journal.pcbi.1013540 (PMC12500091; doi:10.1371/journal.pcbi.1013540)
Supplement: S1 Text — Supplementary Note A in S1 Text. The LSTM model. Provides detailed description of LSTM, with Equations S1–S6. Supplementary Note B in S1 Text. Discretized compartmental model by Euler method. Provides detailed description of compartmental model, with Equations S7. Supplementary Note C in S1 Text. Encoding mobility. Provides detailed description of mobility, with Equations S8, S9. Fig A in S1 Text. (a) Spearman correlation coefficients of covariates. (b) p value of correlation coefficients of all the covariates. Fig B in S1 Text. Model selection of climate covariates for different lag groups. The vertical axis shows MAE values for different lag models described in the model selection section, calculated as the average result across all districts. Fig C in S1 Text. Cases prediction for each integrated districts. Y axis is the cases and X axis is time in weeks. Orange line represents LSTM, pink line represents hybrid model and dark points (label) represents newly reported cases data. Fig D in S1 Text. Mean Absolute Error (MAE) calculated on validate data over all the districts for the comparison of with and without strain shift. Y axis is the average MAE of newly reported cases over all the districts. MAE over all time steps of the model introducing the shifting strain(model V) and the model without introducing shifting strain(model IV) are 491 and 519. Fig E in S1 Text. Mean Absolute Error (MAE) calculated on validate data over all the districts. Y axis is average MAE of newly reported cases over all the districts. MAE over all time steps of the model introducing the shifting strain and the model without introducing shifting strain are 491 and 538. Fig F in S1 Text. Interpretation of important covariates. Each point in the figure represents the change of output of the model at different times and different districts, when the input was increased by 1%. The details of our adapted sensitivity analysis can be found in the method section. The X-axis represents value of co [file pcbi.1013540.s001.docx]

**Supporting Information for**

An explainable covariate compartmental model for predicting the spatio-temporal patterns of dengue in Sri Lanka

Yichao Liu^1^, Peter Fransson^1^, Julian Heidecke^1,2^, Prasad Liyanage^2^, Jonas Wallin^3^, Joacim Rocklöv^1,2, 4^

^1^Interdisciplinary Center for Scientific Computing, Heidelberg, Germany

^2^ Heidelberg Institute of Global Health, Heidelberg University, Germany

^3^Department of statistics, Lund university, Sweden

^4^Department of public health and clinical medicine, Section of sustainable health, Umeå university, Sweden

Yichao Liu

Email: [yichao.liu@iwr.uni-heidelberg.de](mailto:yichao.liu@iwr.uni-heidelberg.de)

**This PDF file includes:**

Supporting text

Figures S1 to S9

Tables S1 to S3

SI References

**list of captions:**

**Supplementary Note A in SI Text.** The LSTM model. Provides detailed description of LSTM, with Equations S1–S6.

**Supplementary Note B in SI Text.** Discretized compartmental model by Euler method. Provides detailed description of compartmental model, with Equations S7.

**Supplementary Note C in SI Text.** Encoding mobility. Provides detailed description of mobility, with Equations S8-9.

**Fig. A in SI Text.** (a) Spearman correlation coefficients of covariates. (b) p value of correlation coefficients of all the covariates.

**Fig. B in SI Text. Model selection of climate covariates for different lag groups.** The vertical axis shows MAE values for different lag models described in the model selection section, calculated as the average result across all districts.

**Fig. C in SI Text. Cases prediction for each integrated districts.** Y axis is the cases and X axis is time in weeks. Orange line represents LSTM, pink line represents hybrid model and dark points (label) represents newly reported cases data.

**Fig. D in SI Text.** **Mean Absolute Error (MAE) calculated on validate data over all the districts for the comparison of with and without strain shift.** Y axis is the average MAE of newly reported cases over all the districts. MAE over all time steps of the model introducing the shifting strain(model V) and the model without introducing shifting strain(model IV) are **491** and **519**.

**Fig. E in SI Text.** **Mean Absolute Error (MAE) calculated on validate data over all the districts.** Y axis is average MAE of newly reported cases over all the districts. MAE over all time steps of the model introducing the shifting strain and the model without introducing shifting strain are **491** and **538**.

**Fig. F in SI Text. Interpretation of important covariates.** Each point in the figure represents the change of output of the model at different times and different districts, when the input was increased by 1%. The details of our adapted sensitivity analysis can be found in the method section. The X-axis represents value of covariates. Y-axis represents the increment by our sensitivity analysis.

**Fig. G in SI Text.** Climate covariates of years from 2011 to 2019 for Colombo

**Fig. H in SI Text.** Map of assumed seroprevalence of Sri Lanka at the start of year 2011.

**Fig. I in SI Text.** The directional impact of covariates on the cases for population percentage of age over 60, mobility, mean temperature, precipitation and mean NDVI.

**Table A in SI Text.** Example of socioeconomic data for Colombo district

**Table B in SI Text.** Population of different integrated districts in 2011 as initial value of S and newly reported cases in the first week of 2011 as initial value of I

**Table C in SI Text.** Seroprevalence estimates of Siri Lanka based on sero-prevalence surveys and model interpolation.

**Supporting Information Text**

**Supplementary Note A: The LSTM model**

LSTM is a kind of recurrent neural network (RNN) used for time series data forecasting. A typical LSTM is composed of 3 parts, forget gate (Equation S1), input gate (Equation

S2), and output gate (Equation S3)

$f_{t}=\sigma(W_{f}x_{t}+U_{f}h_{t-1}+b_{f})$ (S1)

$\iota_{t}=\sigma(W_{\iota}x_{t}+U_{\iota}h_{t-1}+b_{\iota})$ (S2)

$o_{t}=\sigma(W_{o}x_{t}+U_{o}h_{t-1}+b_{o})$ (S3)

$z_{t}=tanh(W_{z}x_{t}+U_{z}h_{t-1}+b_{z})$ (S4)

$c_{t}=f_{t}c_{t-1}+i_{t}z_{t}$ (S5)

$h_{t}=o_{t}tanh($ $c_{t}$) (S6)

where, $x_{t}$ is input features, W and U are weights matrices, and b is bias.

**Supplementary Note B: Discretized compartmental model by Euler method**

$S(t+\Delta t)=S(t)+\Delta t\lambda(t)S(t)$

$$E(t+\Delta t)=E(t)+\Delta t(\lambda(t)S(t)-\omega E(t))$$

$I(t+\Delta t)=I(t)+\Delta t(\omega E(t)-\gamma I(t))$ (S7)

**Supplementary Note C: Encoding mobility**

Mobility is a major driver of dengue transmission. With the help of a radiation model, we calculate proxies for the mobility covariate [^3^](https://www.zotero.org/google-docs/?Ci6iuO). The radiation model assumes the mean number of commuters $F_{i\to j}$ between district $i$ and $j$ is:

$F_{i\to j}=F_{i}\frac{n_{i}n_{j}}{(n_{i}+s_{ij})(n_{i}+n_{j}+s_{ij})}.$ (S8)

Here $n_{i}$ is the population at district $i$, and $s_{ij}$ is the total population in a circle with radius equal to the distance between district $i$ and $j$, and centered at $districtj$ (exclude the population at $i$ and $j$). Finally, $F_{i}$ is the total number of commuters from district $i$. By assuming that $F_{i}$ is proportional to $n_{i}$, we get that:

$F_{i\to j}\propto\frac{n_{i}^{2}n_{j}}{(n_{i}+s_{ij})(n_{i}+n_{j}+s_{ij})}:=\kappa_{i\to j}$ (S9)

We estimate the mobility covariate for a focal district, $i$, as the mean $\kappa_{j\to i}$ of all the neighbouring districts, $j$.

a


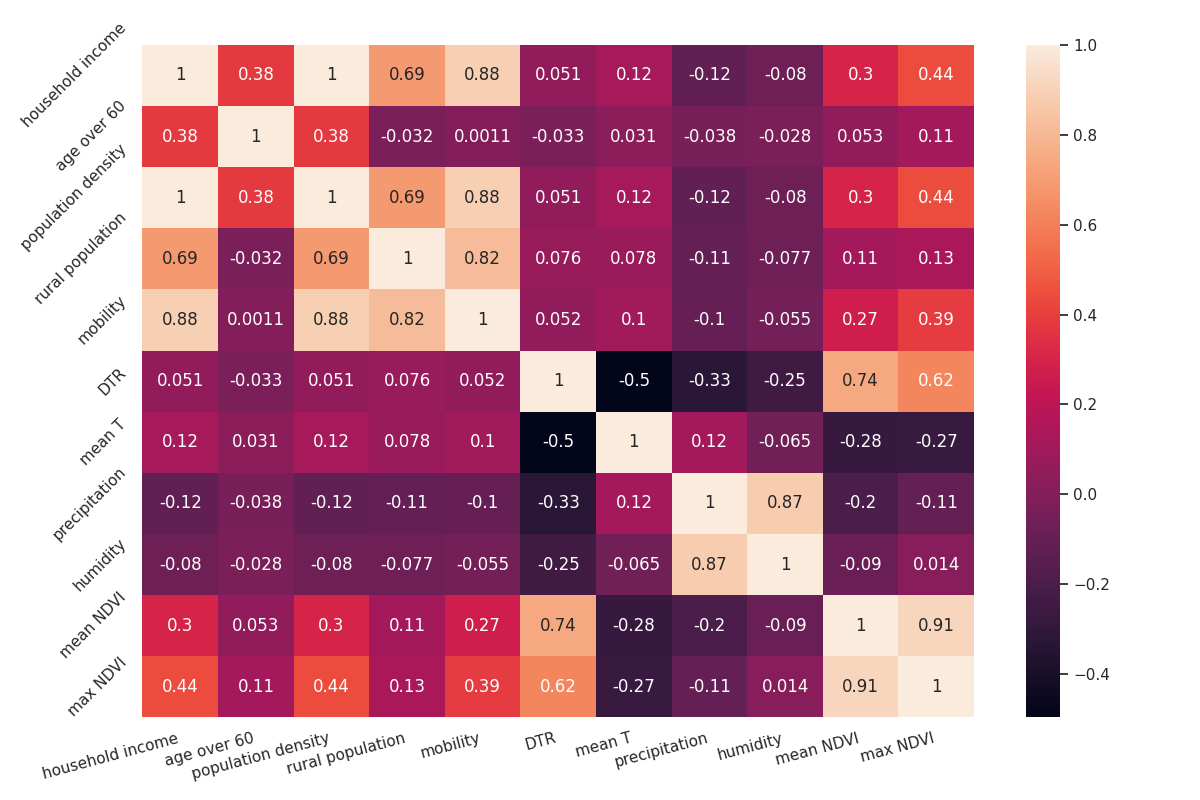


b


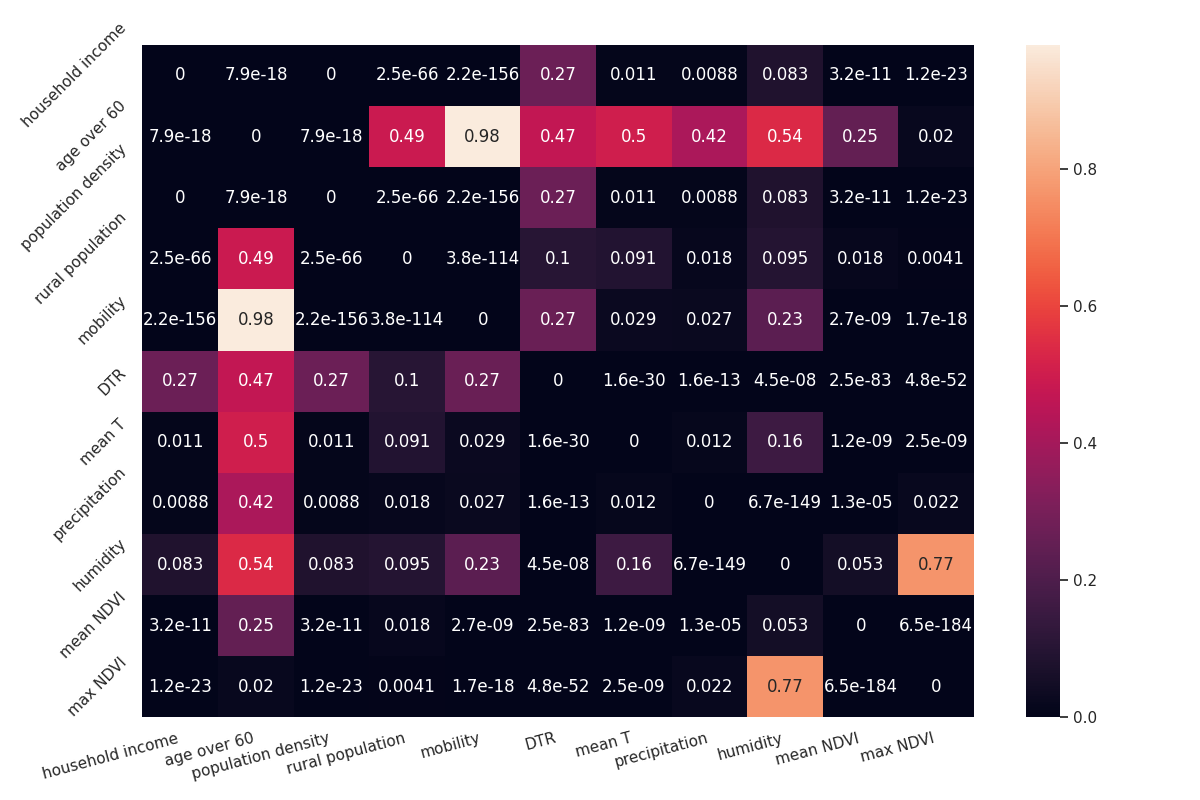


**Fig. A**: (a) Spearman correlation coefficients of covariates. (b) p value of correlation coefficients of all the covariates


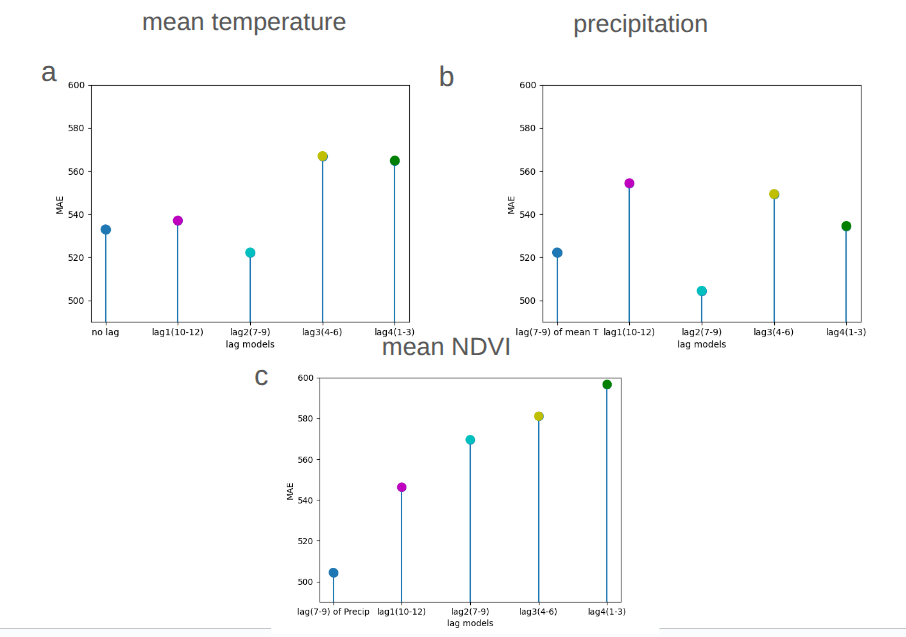


**Fig. B**: Model selection of climate covariates for different lag groups. The vertical axis shows MAE values for different lag models described in the model selection section, calculated as the average result across all districts.


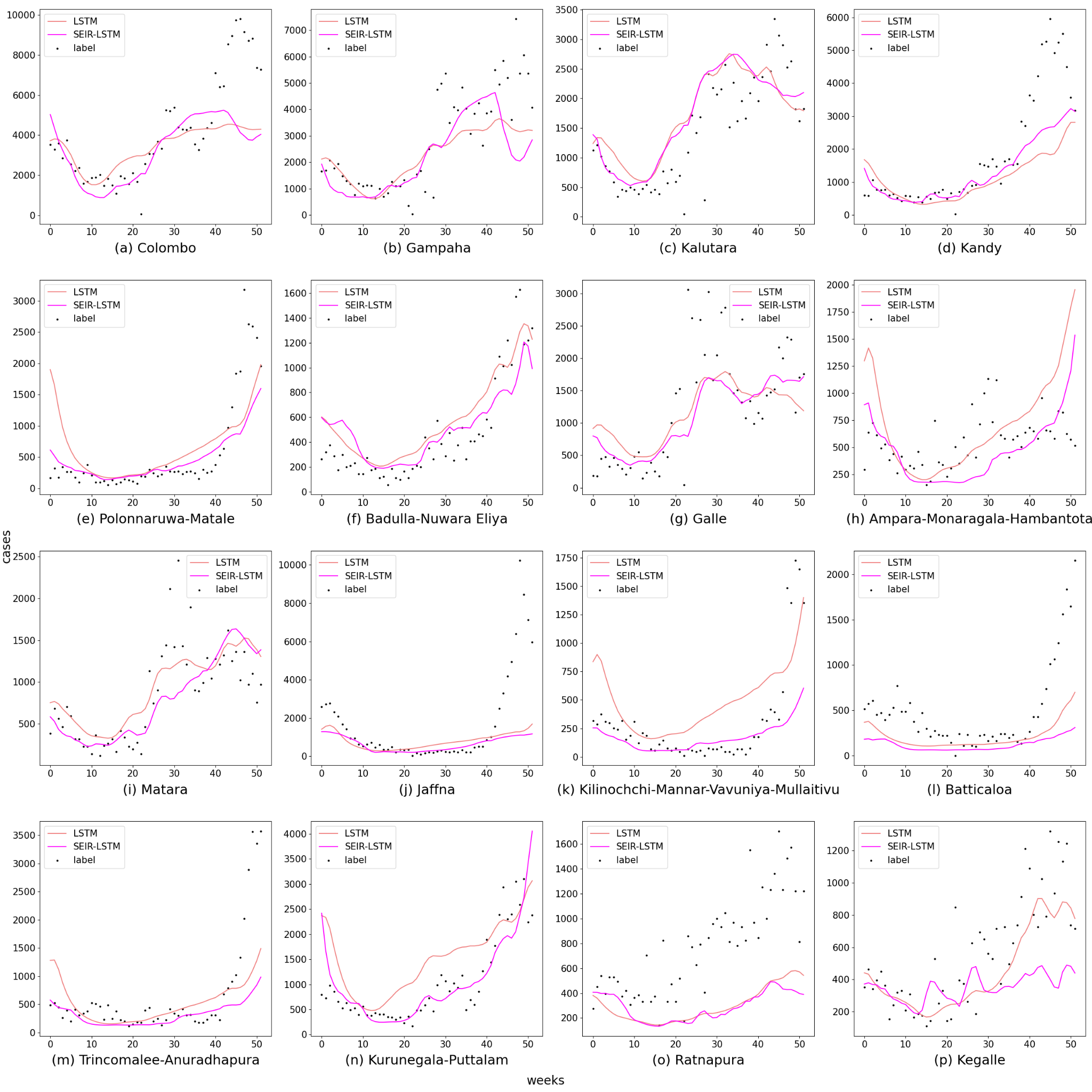


**Fig. C**: Cases prediction for each integrated districts. Y axis is the cases and X axis is time in weeks. Orange line represents LSTM, pink line represents hybrid model and dark points (label) represents newly reported cases data.


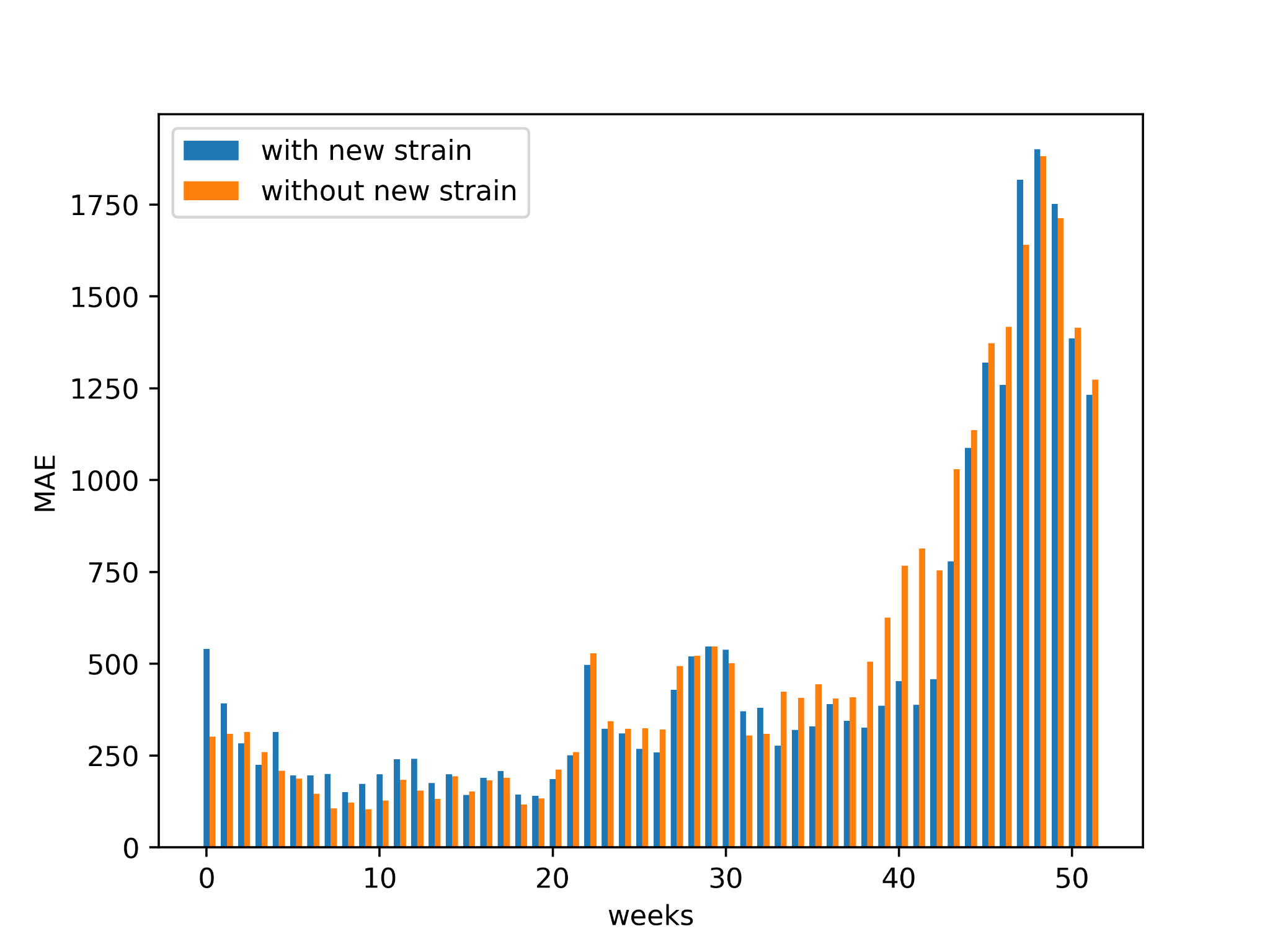


**Fig. D**: Mean Absolute Error (MAE) calculated on validate data over all the districts for the comparison of with and without strain shift. Y axis is the average MAE of newly reported cases over all the districts. MAE over all time steps of the model introducing the shifting strain(model V) and the model without introducing shifting strain(model IV) are **491** and **519**.


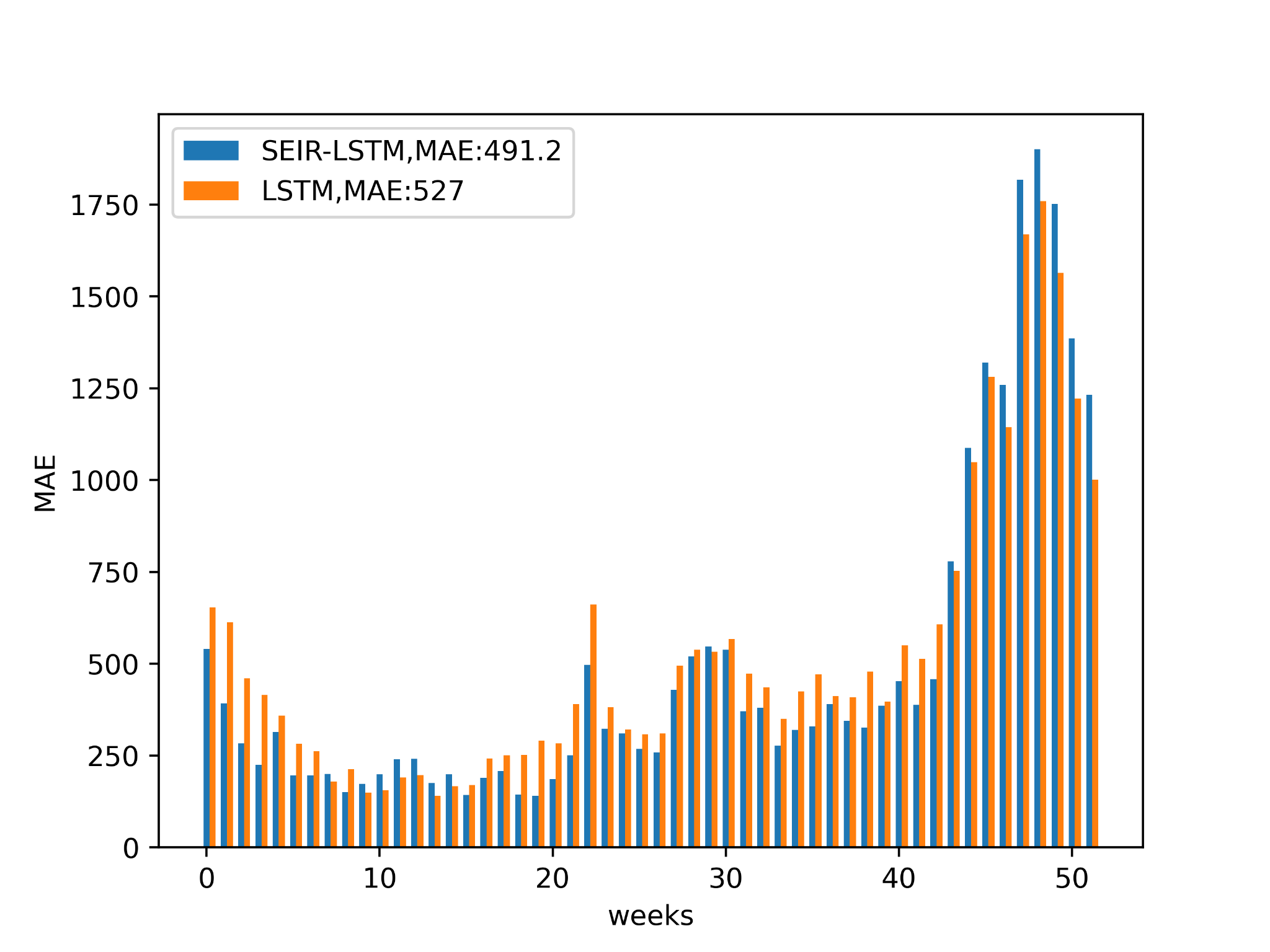


**Fig. E**: Mean Absolute Error (MAE) calculated on validate data over all the districts. Y axis is average MAE of newly reported cases over all the districts. MAE over all time steps of the model introducing the shifting strain and the model without introducing shifting strain are **491** and **538**.


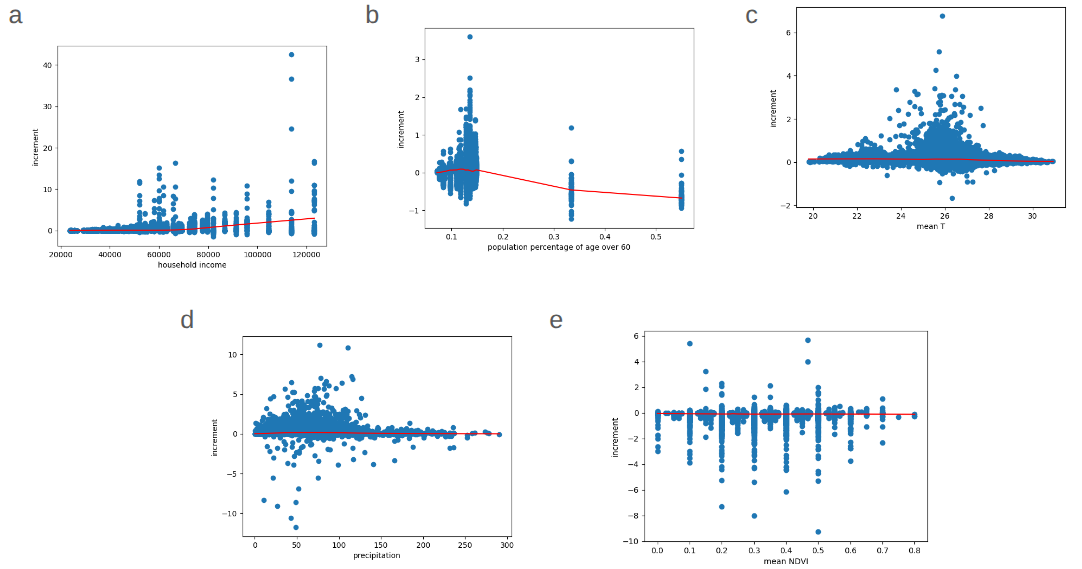


**Fig. F**: Interpretation of important covariates. Each point in the figure represents the change of output of the model at different times and different districts, when the input was increased by 1%. The details of our adapted sensitivity analysis can be found in the method section. The X-axis represents value of covariates. Y-axis represents the increment by our sensitivity analysis.


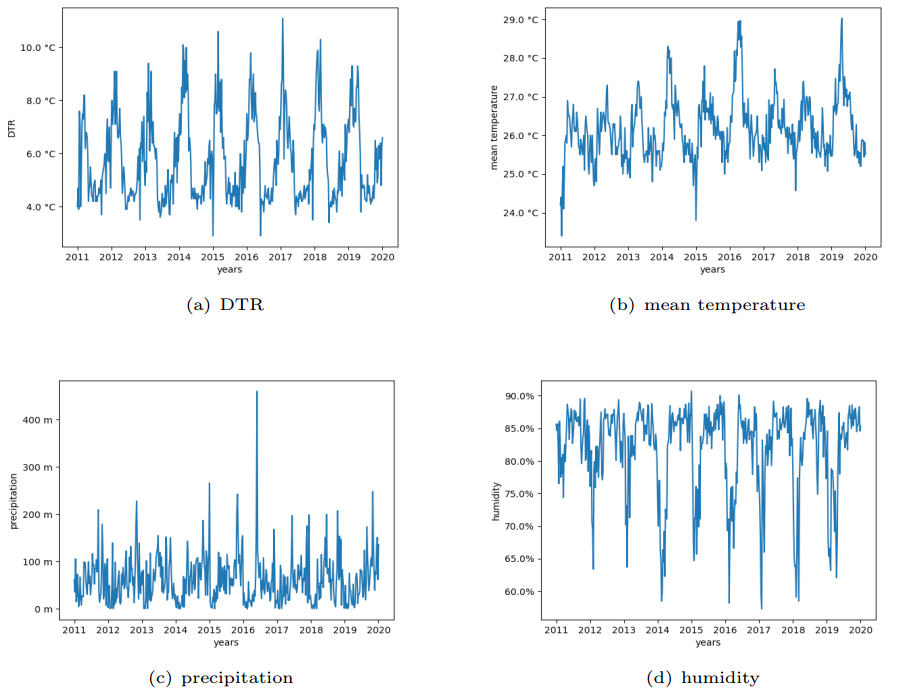

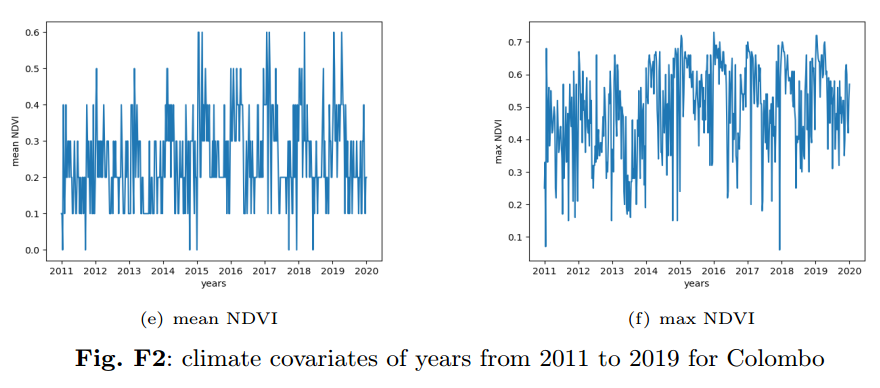


**Fig. G**: Climate covariates of years from 2011 to 2019 for Colombo


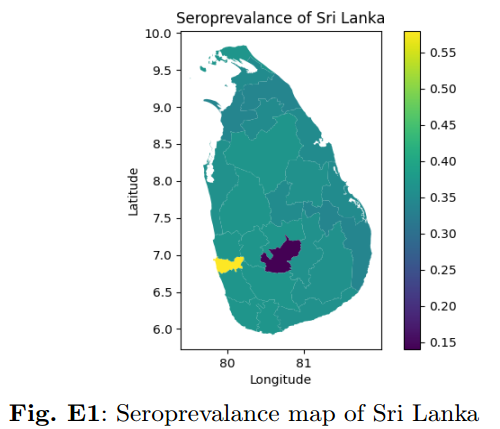


**Fig. H**: Map of assumed seroprevalence of Sri Lanka at the start of year 2011.


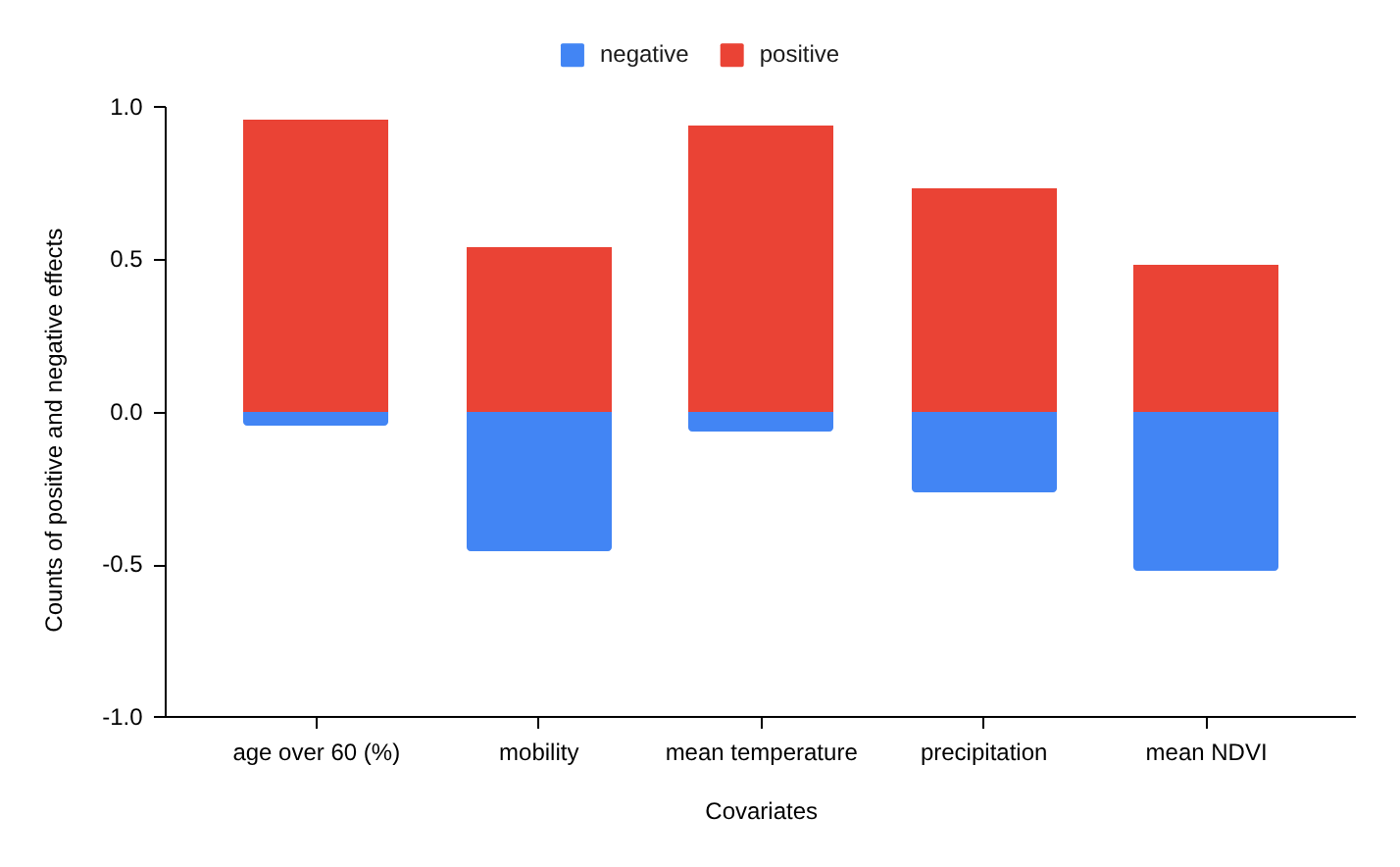


**Fig. I**: The directional impact of covariates on the cases for population percentage of age over 60, mobility, mean temperature, precipitation and mean NDVI.

**Table A**: Example of socioeconomic data for Colombo district

| Colombo | household income (per month) | percentage of age over 60 | population density (per ${km}^{2}$) | rural population | population | mobility |
| --- | --- | --- | --- | --- | --- | --- |
| 2011 | 59954 | 1.143*${10}^{-1}$ | 3304.6 | 669133 | 2309900 | 78956 |
| 2012 | 68838.7 | 1.326*${10}^{-1}$ | 3325 | 635669 | 2324349 | 79563 |
| 2013 | 77723 | 1.359*${10}^{-1}$ | 3325 | 513534 | 2324349 | 79340 |
| 2014 | 86675.7 | 1.359*${10}^{-1}$ | 3362 | 330259 | 2349675 | 80381 |
| 2015 | 95628 | 1.359*${10}^{-1}$ | 3398 | 146983 | 2375000 | 81258 |
| 2016 | 104581 | 1.359*${10}^{-1}$ | 3429 | 529142 | 2396773 | 82122 |
| 2017 | 113865 | 1.359*${10}^{-1}$ | 3460 | 534345 | 2418545 | 83051 |
| 2018 | 123149 | 1.359*${10}^{-1}$ | 3489 | 538820 | 2438799 | 83903 |
| 2019 | 132433 | 1.359*${10}^{-1}$ | 3502 | 540771 | 2447631 | 83988 |

**Table B**: Population of different integrated districts in 2011 as initial value of S and newly reported cases in the first week of 2011 as initial value of I

| districts | Colombo | Gampaha | Kalutara | Kandy | P-M | B-N | Galle | H-A-M |
| --- | --- | --- | --- | --- | --- | --- | --- | --- |
| Susceptible population | 2309900 | 2296666 | 1188000 | 1401000 | 896491 | 1600989 | 1072361 | 1701562 |
| Infectious population | 418 | 132 | 33 | 11 | 88 | 66 | 22 | 77 |

| districts | Matara | Jaffna | K-M-V-M | Batticaloa | T-A | K-P | Ratnapura | Kegalle |
| --- | --- | --- | --- | --- | --- | --- | --- | --- |
| Susceptible population | 799688 | 583400 | 500233 | 589706 | 1304629 | 2370075 | 1128481 | 836603 |
| Infectious population | 22 | 99 | 33 | 33 | 110 | 231 | 33 | 77 |

**Table C**: Seroprevalence estimates of Siri Lanka based on sero-prevalence surveys and model interpolation.

| districts | Colombo | Gampaha | Kalutara | Kandy | Matale | Nuwara Eliya | Galle | Matara |
| --- | --- | --- | --- | --- | --- | --- | --- | --- |
| immunity level | 0.58 | 0.37 | 0.37 | 0.37 | 0.35 | 0.14 | 0.37 | 0.37 |

| districts | Hambantota | Jaffna | Mannar | Vavuniya | Mullaitivu | Killnuchchi | Batticaloa | Ampara | Trincoumake |
| --- | --- | --- | --- | --- | --- | --- | --- | --- | --- |
| immunity level | 0.36 | 0.37 | 0.34 | 0.35 | 0.34 | 0.35 | 0.35 | 0.34 | 0.35 |

| districts | Kurunigala | Puttalam | Amuradhapura | Polomaruwa | Badulla | Monaragala | Ratnapura | Kegalle |
| --- | --- | --- | --- | --- | --- | --- | --- | --- |
| immunity level | 0.37 | 0.36 | 0.37 | 0.36 | 0.36 | 0.36 | 0.37 | 0.37 |

The seroprevalence of Sri Lanka districts was extrapolated from the observed relationship from the works[^1,2^](https://www.zotero.org/google-docs/?qRyuoW) using a regression model. The model assumed the seroprevalence was a function of mean age and climate region using a quasi Poisson distribution function.

**SI References**

[1. Ster, I. C. *et al.* Age-dependent seroprevalence of dengue and chikungunya: inference from a cross-sectional analysis in Esmeraldas Province in coastal Ecuador. *BMJ Open* **10**, e040735 (2020).](https://www.zotero.org/google-docs/?ShDiYW)

[2. Low, S.-L. *et al.* Dengue seroprevalence of healthy adults in Singapore: serosurvey among blood donors, 2009. *Am. J. Trop. Med. Hyg.* **93**, 40 (2015).](https://www.zotero.org/google-docs/?ShDiYW)

[3. Simini, F., González, M. C., Maritan, A. & Barabási, A.-L. A universal model for mobility and migration patterns. *Nature* **484**, 96–100 (2012).](https://www.zotero.org/google-docs/?ShDiYW)
